# Socioeconomic status, health-related behaviours, and death among older people: the Concord health and aging in men project prospective cohort study

**DOI:** 10.1186/s12877-020-01648-y

**Published:** 2020-07-29

**Authors:** Saman Khalatbari-Soltani, Fiona M. Blyth, Vasi Naganathan, David J. Handelsman, David G. Le Couteur, Markus J. Seibel, Louise M. Waite, Erin Cvejic, Robert G. Cumming

**Affiliations:** 1 The University of Sydney School of Public Health, Faculty of Medicine and Health, Sydney, New South Wales Australia; 2 ARC Centre of Excellence in Population Aging Research (CEPAR), University of Sydney, Sydney, Australia; 3 Concord Clinical School, Faculty of Medicine and Health, University of Sydney, Sydney, NSW Australia; 4 Centre for Education and Research on Ageing, Faculty of Medicine and Health, University of Sydney, Sydney, New South Wales Australia; 5 Ageing and Alzheimer’s Institute, Concord Repatriation and General Hospital, Sydney Local Health District, Sydney, New South Wales Australia; 6 ANZAC Research Institute, University of Sydney and Concord Hospital, Sydney, Australia

**Keywords:** Socioeconomic issues, Successful aging, Epidemiology

## Abstract

**Background:**

Conflicting evidence exists regarding the association of socioeconomic status (SES) with mortality among older people and little is known about the mechanisms underlying this association. We investigated the association of SES with mortality among older Australian men. We also investigated potential mediating effects of health-related behaviours in SES-mortality associations.

**Methods:**

We used data from a prospective population-based cohort (the Concord Health and Aging in Men Project), in Sydney, Australia. The main outcomes were all-cause and cause-specific mortality. Educational attainment, occupational position, source of income, housing tenure, and a cumulative SES score were assessed at baseline. Longitudinally assessed alcohol consumption, smoking, physical activity, and body mass index were investigated as potential mediators. Associations were quantified using Cox regression.

**Results:**

We evaluated 1527 men (mean age: 77.4 ± 5.5 years). During a mean follow-up time of 9.0 years, 783 deaths occurred. For deaths from all causes, the adjusted hazard ratio (HR) for the lowest tertile of cumulative SES score versus the highest tertile was 1.44 (95% CI 1.21 to 1.70); the corresponding sub-HRs were 1.35 (0.96 to 1.89) for cardiovascular disease (CVD) mortality; 1.58 (1.15 to 2.18) for cancer mortality, and 1.86 (1.36 to 2.56) for non-CVD, non-cancer mortality. SES-mortality associations were attenuated by 11–25% after adjustment for mediating health-related behaviours.

**Conclusion:**

Low SES is associated with increased mortality in older Australian men and health-related behaviours accounted for less than one-fourth of these associations. Further research is needed to fully understand the mechanisms underlying SES inequalities in mortality among older people.

## Background

Socioeconomic inequalities in mortality is a major public health issue. Although people are living longer than before, socioeconomic inequalities in mortality continue to grow, both globally and in Australia [1, 2]. Mounting evidence suggests that socioeconomic status (SES) is one of the strongest predictors of deaths from all and specific causes [3]. However, the evidence for the association between SES and mortality, which is a core indicator for public health is equivocal in older populations, with some previous studies finding an SES-mortality gradient at older ages [4–7] and other studies finding no association [8–10]. Most studies of socioeconomic inequalities in mortality have relied on a single indicator of SES, mainly education, and have examined only all-cause mortality or one cause of death at a time [5].

Behavioural risk factors, such as heavy drinking, smoking, physical inactivity, and unhealthy diet, have been proposed as one of the underlying pathways explaining the SES gradient in mortality [11]. These risk factors tend to be more frequent among disadvantaged SES groups [12, 13] and so health-related behaviours may mediate the association between SES and mortality. Among middle-aged individuals, a recent systematic review of observational studies showed that health-related behaviours explained 20 to 26% of SES inequalities in mortality [14]. However, among older individuals, little evidence, with mixed results, exists regarding the mediating impact of health-related behaviours on SES inequalities in mortality [15–18]. The inconsistency among these studies may be explained by the different SES indicators used, as well as differences in participant characteristics and whether health-related behaviours were assessed using single or longitudinal measures. Indeed, previous studies have shown that the mediating impact of health-related behaviours differed by age [15], study design [3], and location [19].

The Australian population is ageing. In 2017, 15% of Australian (3.8 million) were aged 65 and over and 66% of deaths were among people aged 75 and over [20]. Australia has a universal health care system; however, this is insufficient in itself to reduce health inequalities. England, similarly has universal health service coverage, but widespread and large health inequalities exist [21]. Moreover, although nearly two-thirds of older Australian receive government assistance as their main source of income, many are at risk of poverty [22]. Thus, to reduce health inequalities, we need to improve the conditions in which people are born, live, work, and age.

The first objective of our study was to investigate the association between SES and all-cause and cause-specific mortality over a 9-year follow-up period using data from a population-based cohort of older Australian men. We used educational level, occupational position, source of income, and housing tenure as well a cumulative SES score from early adult life to older age as indicators of SES. The second objective was to assess the potential mediating effect of health-related behaviours in the association between SES and mortality.

## Methods

### Study population

The Concord Health and Aging in Men Project (CHAMP) is an on-going population-based cohort of men aged 70 years and older [23]. Men were a particular focus of the CHAMP study as to date epidemiological studies of ageing have tended to focus on women [23]. Briefly, men living in a defined urban geographical region (the Local Government Areas of Burwood, Canada Bay, and Strathfield) near Concord Hospital in the city of Sydney, Australia were recruited [23]. The sampling frame was the New South Wales Electoral Roll, on which registration is compulsory. The only exclusion criteria was living in an aged care facility. There were four study phases: recruitment in 2005–2007 (*n* = 1705), the first follow-up in 2007–2009 (*n* = 1366), the second follow-up in 2012–2013 (*n* = 954), and the third follow-up in 2015–2016 (*n* = 779) [24]. The initial baseline participation rate was 54% among eligible men with whom contact was made. The CHAMP study complied with the World Medical Association Declaration of Helsinki and was approved by the Central Sydney Area Health Service Human Research Ethics Committee. Written informed consent was obtained from all the participants involved in the study.

### Socioeconomic indicators

Four self-reported indicators of SES related to early adult life and to older ages were used: educational attainment, occupational level, source of income, and housing tenure. Educational attainment is an SES measure that includes both early life and young adulthood [25]. Occupational position is the most commonly used measure of adult SES [25]. Source of income and housing tenure are measures of current SES [25].

Educational attainment was assessed based on the highest qualification attained and categorized as ‘high’ (university degree), ‘intermediate’ (trade, apprenticeship, certificate, or diploma), and ‘low’ (no post-school qualification).

Occupational position was based on longest occupation held during working life. Occupational position was first classified into eight major groups based on the Australian and New Zealand Standard Classification of Occupations (ANZSCO) and three main categories were subsequently defined: ‘high’ (higher professional and managers, lower professionals and managers, higher clerical services and sales workers), ‘intermediate’ (small employers and self-employed, farmers, lower supervisors, and technicians) and ‘low’ (lower clerical, services, sales workers, skilled and unskilled workers) [26].

Australia’s retirement income system comprises “three pillars”: a means-tested age pension, mandatory occupational superannuation, and voluntary long-term savings [27]. Participants reported their sources of income by selecting from the following six response options (more than one response allowed): ‘age pension’, ‘repatriation pension/veteran’s pension’, ‘superannuation or other private income’, ‘own business/farm/partnership’, ‘wage or salary’, and ‘other (please specify)’. Subsequently, source of income was categorized into ‘high’ (sources of income do not include any no government pension), ‘intermediate’ (reliant on a government pension *plus* other sources of income), and ‘low’ (solely reliant on a government pension).

Housing tenure was assessed based on participants’ response to the question about their housing arrangement and categorized as ‘owner’ (owning home outright), and ‘other’ (e.g. leasing or purchasing in a retirement village, paying rent to a private landlord housing, and paying rent to the government for public housing). Owner-occupiers were specified as higher SES.

One of the conceptual models describe the impact of SES on health in adulthood is the cumulative effect of exposure to adverse SES from across the life course that affect health in a dose-response manner [28]. To assess the dose-response of cumulative exposure to low SES [28], we calculated a cumulative SES score from baseline data [29]. To calculate this score, educational attainment, occupational position, and source of income were coded 0 (high), 1 (intermediate), or 2 (low) and housing tenure was coded 0 (owners) or 1 (other). Then, the four SES indicators were summed (range 0–7) and the final cumulative SES score was divided into tertiles: ‘high’ (score 0–3), ‘intermediate’ (score 4), and ‘low’ (score 5–7).

### Health-related Behaviours

Health-related behaviours were assessed by self-administered questionnaires at baseline and all subsequent follow-up visits. Smoking was categorized as ‘never smoker’, ‘former smoker’, and ‘current smoker’. Alcohol consumption was assessed by using questions on the number of days and number of alcohol units consumed in the past year, then converted to number of alcohol units consumed per week and categorized as ‘abstainer’ (0 units/week during the past year), ‘moderate drinker’ (1–21 units/week), or ‘heavy drinker’ (> 21 units/week) [30]. Physical activity was measured using the Physical Activity Scale for the Elderly (PASE) [31]. The total PASE score was computed by multiplying the amount of time spent in each activity (hours/week) or participation in an activity (yes/no) by empirically derived item weights and summing overall activities. The calculated PASE score was dichotomized at the lowest quartile (< 79 vs ≥80) as the distribution was highly positively skewed.

Body weight and height were measured with participants barefoot and in light indoor clothes at baseline and all follow-ups; body mass index (BMI) was calculated as weight (kg) divided by height squared (m^2^) and was categorized as normal or underweight (BMI < 25 kg/m^2^), overweight (25 ≤ BMI < 30 kg/m^2^), and obese (BMI ≥ 30 kg/m^2^).

### Outcome ascertainment

Consenting participants (*n* = 1639, 96%) were successfully traced and followed up for all-cause mortality through the New South Wales Registry of Births, Deaths and Marriages (RBDM), which records all deaths in New South Wales. Mortality follow-up was available from January 1, 2005 up to December 31, 2017. The cause of death was obtained from the RBDM Cause of Death Unit Record File up to December 31, 2015. Mortality data were linked to the CHAMP baseline data by the Centre for Health Record Linkage (http://www.cherel.org.au/) using probabilistic record linkage methods and Choice-Maker software.

Deaths from all-cause, cardiovascular disease (CVD), cancer, and non-CVD, non-cancer were examined. The International Classification of Diseases -10^th^ revision (ICD-10) codes were used to define CVD mortality (I00-I99), and cancer mortality (C00-C97). All remaining deaths were grouped as ‘non-CVD, non-cancer’ mortality, with the most common causes being diseases of respiratory system (J00-J99), diseases of nervous system (G00-G99), and external causes (V00-Y98).

### Assessment of covariates

We included age (continuous), living arrangement (living with others/living alone), and country of birth (Australian-born/other) as potential confounders. In this study, we did not control for high blood pressure, hypercholesterolemia, and diabetes because previous research among CHAMP men found no association between these traditional risk factors and mortality [32]. Notably, we decided a priori not to adjust for risk factors of mortality including comorbid conditions, quality of life, access to health care because they are likely to be on the causal pathway of the association between SES and mortality. For instance, low SES is associated with poor quality of life and presence of comorbid conditions which in turn increase the risk of mortality [1]. Thus, adjustment for risk factors of mortality may represents an over adjustment and will not reflect an overall effect of SES and mortality [33].

### Statistical analysis

Statistical analyses were performed using Stata (version 15; StataCorp, College Station, TX, USA). Of the 1527 participants with information on health-related behaviours and BMI at baseline, 939 participants had complete data on all health-related behaviours and BMI at follow-ups before death occurred (Table S1). We accounted for missing observations at each time point for both non-response and non-responders for health-related behaviours and BMI using chained equations [34]. Missing values on SES indicators, our exposure variables, were not imputed. Ten imputed datasets were generated and analysed. The imputation model included age, all confounding and mediating variables, and survival status. We assessed the validity of our imputation model by comparing the distribution of complete data with imputed datasets. Similar distributions of imputed variables with the complete data suggests that the imputed model fitted properly.

For each individual SES indicator and each tertile of cumulative SES score, mortality rates per 1000 person-years were calculated for all-cause, and cause-specific mortality. These rates were standardized for age (5-year age groups) with the direct method using the 2017 New South Wales population as reported by the Australian Bureau of Statistics (http://stat.data.abs.gov.au).

Cox proportional-hazards regression was used to examine the associations between baseline health-related behaviours and BMI and all-cause mortality. Associations with cause-specific mortality were examined using Fine and Gray’s competing-risks survival regression (proportional sub-hazards model) [35]. Adjusted hazard ratios (HRs) or sub-hazard ratios (SHRs) and 95% confidence intervals (CIs) are reported.

Cox proportional-hazards regression was also used to assess the associations between baseline individual SES indicators and tertiles of cumulative SES scores as independent variables and all-cause mortality as dependent variables. Competing-risk survival regression was used for cause-specific mortality. Survival time was measured as the time from the date of baseline interview to either the date of death, or end of follow-up (December 31, 2017 for all-cause mortality; December 31, 2015 for cause-specific mortality). The proportional hazards assumption was assessed using Schoenfeld residuals; in all models this assumption was satisfied. All Cox proportional-hazard regression models were adjusted for age, living arrangement, and country of birth (reference model). Linear trends were assessed by using orthogonal polynomial contrasts. Subsequently, longitudinally assessed alcohol consumption, smoking, physical activity, and BMI were entered individually and then simultaneously as time-varying covariates into a reference model with the baseline cumulative SES scores as the exposure variable and mortality as the outcome variable. HRs were calculated for the lowest versus highest tertile of cumulative SES score. All covariates were entered as categorical variables, except age.

The mediating effect of each risk factors on the cumulative SES-mortality association was determined by the percent attenuation in the β coefficient [36] for SES after inclusion of each risk factor one at a time and then simultaneously into our reference model (“change-in-estimate” method) by using the following formula: 100 * (β_Model 1_-β_Model 1 + health behaviour(s)_)/ (β_Model 1_), as previously conducted [18]. This analysis of attenuation was assumed to have no interaction between exposure and mediators [37]. We therefore tested for interaction between each indicator of SES and health-related behaviours on the risk of mortality. We also controlled for potential mediator-outcome confounders (age, living arrangement, and country of birth).

A priori, we examined whether the associations between individual SES measures and cumulative SES scores and mortality varied by age, testing for statistical interaction by age and conducting analysis stratified by age (70–79 and 80+).

### Sensitivity analyses

Mediation analyses were repeated in subgroups including participants with complete data (complete-case analysis), to identify possible source of bias. Furthermore, to investigate the potential influence of reverse causality, that is, baseline presence of diagnosed and undiagnosed comorbid conditions raising the short-term risk of mortality, which may influence SES, we repeated the analyses after excluding participants who died during the first two years of follow-up. This sensitivity analysis also helps control for end of life declines in health behaviours [15].

## Results

Out of 1705 participants at baseline, we excluded those who did not agree to mortality data linkage (*n* = 66), and those who had missing data for SES (*n* = 55), baseline health-related behaviours (n = 55), or covariates (*n* = 2). After excluding these participants, 1527 men (mean age: 77.4 ± 5.5 years) were available for analyses (Figure S1). Participants included were more likely to be Australian-born and physically active in comparison to excluded individuals (Table S2). There were no important differences for age, living arrangement, alcohol consumption, smoking, or BMI.

Table 1 shows baseline characteristics of participants according to SES. Participants in the low SES group were more likely to be non-Australian-born than participants in the high SES group. Overall, those in low SES groups were more likely to be a current smoker and be physically inactive but were less likely to be harmful alcohol drinkers. Over the study follow-up period, the prevalence of smoking declined while the prevalence of physical inactivity increased in both the highest and lowest cumulative SES tertile categories (Figure S2).

**Table 1:** Characteristics of participants by indicators of socioeconomic status at baseline, the CHAMP study

Characteristics	Education			Occupation			Income			Housing tenure		Tertile groups of cumulative SES		
	High (*n* = 185)	Intermediate (*n* = 654)	Low (*n* = 688)	High (*n* = 464)	Intermediate (*n* = 578)	Low (*n* = 485)	High (*n* = 686)	Intermediate (*n* = 255)	Low (*n* = 586)	Owner (*n* = 1368)	Other (*n* = 159)	High (*n* = 769)	Intermediate (*n* = 319)	Low (*n* = 439)
Age, years	77.1 ± 5.1	77.3 ± 5.5	77.6 ± 5.5	77.5 ± 5.5	77.4 ± 5.7	77.4 ± 5.3	77.6 ± 5.7	77.2 ± 5.3	77.3 ± 5.2	77.5 ± 5.5	77 ± 5.5	77.4 ± 5.6	77.9 ± 5.6	77.1 ± 5.1
Age categories, %														
70–79 (*n* = 1085)	71.4	72.1	70.1	70.4	72.5	70.1	66.9	74.9	74.4	70.2	79.1	70.1	67.4	75.6
80+ (n = 441)	28.6	27.9	29.9	29.6	27.5	29.9	33.1	25.1	25.6	29.8	20.9	29.9	32.6	24.4
Country of birth, %														
Australian-born (*n* = 780)	64.9	59.6	39.2	68.1	46.0	40.8	63.1	63.5	31.6	51.3	49.1	64.6	49.8	28.2
Other (*n* = 747)	35.1	40.4	60.8	31.9	54.0	59.2	36.9	36.5	68.4	48.7	50.9	35.4	50.2	71.8
Marital status, %														
Single (n = 76)	7.0	4.4	4.9	5.2	5.2	4.5	4.8	5.5	4.9	4.2	11.3	4.7	6.6	4.3
Married/Defacto (*n* = 1175)	81.1	75.5	77.2	79.7	73.4	78.6	78.6	76.9	75.1	79.8	52.8	79.3	73.0	75.6
Widowed/divorced (*n* = 276)	11.9	20.0	17.9	85.1	78.0	81.6	82.2	79.2	81.2	16.0	35.8	16.0	20.4	20.0
Living alone, % (*n* = 285)	17.3	20.0	17.7	14.9	22.0	18.4	17.8	20.8	18.8	17.0	33.3	17.4	20.7	19.4
Alcohol, %														
Abstainer (*n* = 353)	27.6	21.1	23.8	20.7	23.4	25.2	20.6	22.0	26.6	22.1	31.4	20.9	24.5	26.0
Moderate drinker (*n* = 1053)	65.4	71.3	67.7	70.3	68.3	68.5	70.6	67.8	67.6	70.0	60.4	70.2	68.3	67.2
Heavy drinker (*n* = 121)	7.0	7.6	8.4	9.1	8.3	6.4	8.9	10.2	5.8	7.9	8.2	8.8	7.2	6.8
Smoking, %														
Non-smoker (*n* = 562)	49.7	38.4	31.8	43.5	34.8	32.8	40.7	36.1	32.6	36.8	37.1	42.0	37.0	27.6
Ex-smoker (*n* = 873)	47.6	57.0	59.9	51.5	59	60.4	56.0	60.4	57.2	57.5	54.7	54.5	57.1	62.0
Current smoker (*n* = 92)	2.7	4.6	8.3	5.0	6.2	6.8	3.4	3.5	10.2	5.8	8.2	3.5	6.0	10.5
Physical activity, %														
Active (*n* = 1161)	72.4	80.1	73.1	75.6	77.2	75.1	77.3	76.9	74.2	78.1	57.9	78.2	75.5	72.7
Inactive (*n* = 366)	27.6	19.9	26.9	24.4	22.8	24.9	22.7	23.1	25.8	21.9	42.1	21.8	24.5	27.3
BMI, kg/m^2^	26.7 ± 3.9	27.7 ± 3.9	28.1 ± 4.1	27.3 ± 3.7	27.7 ± 3.9	28.2 ± 4.4	27.7 ± 3.7	27.4 ± 4.3	28 ± 4.2	27.8 ± 3.8	27.4 ± 5.5	27.4 ± 3.8	28.2 ± 3.9	28.2 ± 4.4
BMI categories, %														
Underweight/normal (*n* = 370)	34.1	22.9	22.8	28.2	22.1	22.9	23.5	28.2	23.4	23.2	32.7	26.8	19.1	23.5
Overweight (*n* = 748)	47.6	52.1	46.4	46.8	52.6	46.8	49.9	48.2	48.3	50.1	39.6	49.2	53.3	45.6
Obese (*n* = 409)	18.4	24.9	30.8	25.0	25.3	30.3	26.7	23.5	28.3	26.7	27.7	24.1	27.6	31.0

Abbreviation: SES, socioeconomic status; BMI, body mass index

*N* = 1527. Data are mean ± SD for continuous variables or percent for categorical variables, unless otherwise stated

Educational attainment was categorized as ‘high’ (university degree), ‘intermediate’ (trade, apprenticeship, certificate, or diploma), and ‘low’ (no post-school qualification); Occupational level was categorized as ‘high’ (higher professional and managers, lower professionals and managers, higher clerical services and sales workers), ‘intermediate’ (small employers and self-employed, farmers, lower supervisors, and technicians), and ‘low’ (lower clerical, services, sales workers, skilled and unskilled workers); and source of income was categorized as ‘high’ (other sources of income only), ‘intermediate’ (reliant on a government pension plus other source of income), and ‘low’ (reliant on a government pension only)

During a mean (SD) follow-up time of 9.0 (3.5) years, 783 deaths from all causes occurred (Table 2). There were 197 deaths from CVD, 215 from cancer, and 218 from non-CVD, non-cancer causes during a mean 8.0 (SD: 2.8) years of follow-up (Table 2). There was a social gradient by educational level for all-cause and CVD-mortality, by source of income for cancer and non-CVD, non-cancer mortality, and by housing tenure for all-cause and non-CVD, non-cancer mortality. A clear social gradient in all-cause and cause-specific mortality was evident across tertile groups of cumulative SES (Table 2).

**Table 2:** Age-standardized mortality rates as a function of socioeconomic status, the CHAMP study

	All-cause mortality			Cause-specific mortality						
	Person years of follow-up ^b^	n	Rate ^c^ (95% CI)	Person years of follow-up ^d^	CVD Mortality		Cancer Mortality		Other Mortality ^a^	
					n	Rate ^c^ (95% CI)	n	Rate ^c^ (95% CI)	n	Rate ^c^ (95% CI)
Overall (*n* = 1527)	13,814	783	55.3 (51.4 to 59.1)	12,180	197	16.6 (14.3 to 18.9)	215	15.7 (13.6 to 17.8)	218	18.0 (15.6 to 20.5)
Education										
High (*n* = 185)	1808	80	41.2 (32.1 to 50.2)	1580	16	9.3 (4.8 to 13.9)	22	11.9 (6.9 to 16.9)	21	13.4 (7.7 to 19.2)
Intermediate (n = 654)	5969	336	57.6 (51.4 to 63.7)	5264	75	15.2 (11.7 to 18.6)	96	17.1 (13.7 to 20.6)	93	20.0 (15.9 to 24.0)
Low (*n* = 688)	6038	367	58.4 (52.4 to 64.4)	5336	106	20.2 (16.3 to 24.0)	97	15.9 (12.8 to 19.1)	104	18.5 (14.9 to 22.0)
Occupational position										
High (*n* = 464)	4342	223	46.3 (40.2 to 52.3)	3811	51	12.5 (9.0 to 15.9)	57	13.8 (10.2 to 17.4)	62	14.5 (10.9 to 18.1)
Intermediate (*n* = 578)	5111	307	60.1 (53.4 to 66.8)	4513	80	19.7 (15.4 to 24.0)	83	16.1 (12.6 to 19.6)	88	20.2 (16.0 to 24.4)
Low (*n* = 485)	4362	253	61.3 (53.7 to 68.8)	3856	66	17.8 (13.5 to 22.0)	75	17.5 (13.6 to 21.5)	68	20.3 (15.5 to 25.2)
Source of Income										
High (*n* = 686)	6457	315	48.4 (43.0 to 53.7)	5644	86	16.1 (12.7 to 19.5)	79	12.8 (10.0 to 15.7)	81	14.0 (11.0 to 17.1)
Intermediate (n = 255)	2236	147	63.0 (52.8 to 73.2)	1994	37	17.5 (11.9 to 23.2)	37	16.3 (11.0 to 21.5)	39	19.9 (13.7 to 26.2)
Low (*n* = 586)	5121	321	60.5 (53.8 to 67.1)	4542	74	16.7 (12.9 to 20.5)	99	18.7 (15.0 to 22.4)	98	22.4 (18.0 to 26.8)
Housing tenure										
Owners (*n* = 1368)	12,397	692	54.5 (50.4 to 58.5)	10,914	178	16.8 (14.3 to 19.2)	192	15.7 (13.5 to 17.9)	187	17.2 (14.7 to 19.6)
Other (*n* = 159)	1418	91	63.2 (50.2 to 76.2)	1266	19	15.2 (8.4 to 22.0)	23	16.5 (9.8 to 23.3)	31	26.7 (17.3 to 36.1)
Tertile groups of cumulative SES										
High (0–3, *n* = 769)	7152	372	50.3 (45.2 to 55.5)	6273	94	14.9 (11.9 to 17.9)	96	14.4 (11.5 to 17.3)	95	15.3 (12.2 to 18.3)
Intermediate (4, *n* = 319)	2869	160	56.1 (47.4 to 64.8)	2524	44	18.0 (12.5 to 23.4)	44	15.9 (11.2 to 20.5)	45	16.4 (11.6 to 21.1)
Lowest (5–7, *n* = 439)	3794	251	64.8 (56.8 to 72.8)	3383	61	19.0 (14.2 to 23.7)	75	17.8 (13.7 to 21.8)	78	24.9 (19.4 to 30.4)

Abbreviations: *CVD*, cardiovascular disease; *SES*, socioeconomic status

Educational attainment was categorized as ‘high’ (university degree), ‘intermediate’ (trade, apprenticeship, certificate, or diploma), and ‘low’ (no post-school qualification); Occupational level was categorized as ‘high’ (higher professional and managers, lower professionals and managers, higher clerical services and sales workers), ‘intermediate’ (small employers and self-employed, farmers, lower supervisors and technicians), and ‘low’ (lower clerical, services, sales workers, skilled and unskilled workers); and source of income was categorized as ‘high’ (other sources of income only), ‘intermediate’ (reliant on a government pension plus other source of income), and ‘low’ (reliant on a government pension only)

^a^ Indicates non-cardiovascular disease and non-cancer mortality

^b^ All-cause mortality follow-up was available from January 1, 2005 up to December 31, 2017

^c^ The mortality rates were standardized for age with the direct method using the 2017 New South Wales population based on Australian Bureau of Statistics (http://stat.data.abs.gov.au). The rates are per 1000 person-years

^d^ Cause of deaths follow-up was available from January 1, 2005 up to December 31, 2015

Table 3 shows associations between baseline health-related behaviours and BMI and all-cause and cause-specific mortality. Harmful drinking was not significantly associated with all-cause or cause-specific mortality. Current smoking and physical inactivity were associated with increased all-cause and cause-specific mortality. Being overweight or obese was associated with lower all-cause and cause-specific mortality, except for deaths from cancer. In age-stratified analyses (70 to 79 years versus ≥80 years), the associations between health-related behaviours and mortality were not materially altered, but CIs were wider (Table S3).

**Table 3:** Associations of baseline health behaviours and body mass index with all-cause, and cause-specific mortality, the CHAMP study

	All-cause mortality		CVD-mortality		Cancer-mortality		Other-mortality ^a^	
	n	HR ^b^ (95% CI)	n	SHR ^b^ (95% CI)	n	SHR ^b^ (95% CI)	n	SHR ^b^ (95% CI)
Alcohol consumption								
Abstainer (*n* = 353)	202	Ref.	54	Ref.	54	Ref.	64	Ref.
Moderate drinker (*n* = 1053)	520	0.87 (0.74 to 1.02)	123	0.91 (0.66 to 1.26)	150	0.94 (0.69 to 1.30)	138	0.79 (0.58 to 1.06)
Heavy drinker (*n* = 121)	61	0.97 (0.73 to 1.30)	20	1.50 (0.88 to 2.55)	11	0.58 (0.30 to 1.13)	16	0.83 (0.47 to 1.45)
Smoking								
Non-smoker (*n* = 562)	269	Ref.	82	Ref.	62	Ref.	63	Ref.
Ex-smoker (*n* = 873)	454	1.20 (1.03 to 1.40)	103	0.82 (0.61 to 1.10)	133	1.41 (1.04 to 1.91)	139	1.55 (1.14 to 2.09)
Current smoker (*n* = 92)	60	2.14 (1.61 to 2.85)	12	1.10 (0.57 to 2.10)	20	2.24 (1.34 to 3.75)	16	2.09 (1.20 to 3.64)
Physical activity								
Active (*n* = 1161)	530	Ref.	120	Ref.	161	Ref.	132	Ref.
Inactive (*n* = 366)	253	1.63 (1.39 to 1.90)	77	1.53 (1.14 to 2.07)	54	1.03 (0.75 to 1.43)	86	1.77 (1.31 to 2.39)
BMI categories								
Underweight/normal (*n* = 370)	224	Ref.	67	Ref.	51	Ref.	72	Ref.
Overweight (*n* = 748)	374	0.80 (0.68 to 0.94)	95	0.78 (0.57 to 1.08)	99	0.97 (0.69 to 1.38)	99	0.75 (0.55 to 1.03)
Obese (*n* = 409)	185	0.79 (0.65 to 0.96)	35	0.62 (0.40 to 0.95)	65	1.22 (0.83 to 1.79)	47	0.74 (0.51 to 1.08)

*N* = 1527. Abbreviations: HR, hazard ratio; SHR, sub-hazard ratio; BMI, body mass index

We used calendar year as the time scale, with survivors having a censoring date of 31 December 2017 (person years follow-up = 13,814) for all-cause mortality and with survivors having a censoring date of 31 December 2015 (person years follow-up = 12,180) for cause-specific mortality

^a^ Indicates non-cardiovascular disease and non-cancer mortality

^b^ Adjusted for age, country of birth, and living arrangement

### Indicators of socioeconomic status and mortality

**Fig. 1:**
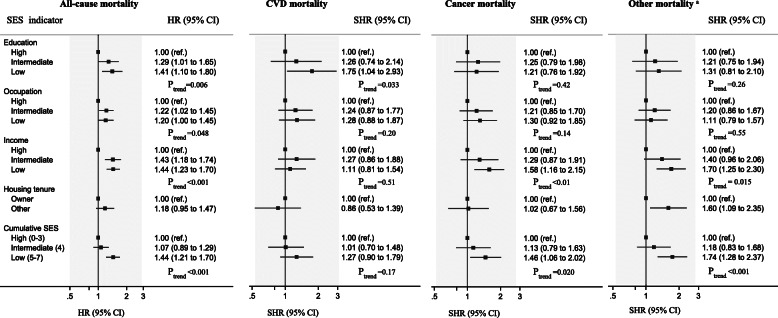
Association of socioeconomic status indicators with all-cause, and cause-specific mortality, the CHAMP study. Abbreviations: CVD, cardiovascular disease; SES, socioeconomic status; HR, hazard ratio; SHR, sub-hazard ratio. *N* = 1527. We used calendar year as the time scale, with survivors having a censoring date of 31 December 2017 (person-years follow-up = 13,814) for all-cause mortality and with survivors having a censoring date of 31 December 2015 (person-years follow-up = 12,180) for cause-specific mortality. All estimates were adjusted for age, country of birth, and living arrangement. ^a^ Indicates non-cardiovascular disease and non-cancer mortality

Figure 1 shows associations between individual SES indicators and tertile categories of cumulative SES score and all-cause and cause-specific mortality. Low educational level was associated with all-cause, and CVD mortality. Being in intermediate or low occupational positions was associated with all-cause mortality, but not cause-specific mortality. There was an inverse dose-response association between source of income and all-cause, cancer, and non-CVD, non-cancer mortality. No association was evident between housing tenure and all-cause or cause-specific mortality, except for deaths from non-CVD, non-cancer. There was an inverse dose-response association across tertiles of cumulative SES and all-cause and cause-specific mortality (P_trend_ < 0.05), except for deaths from CVD (P_trend_ = 0.17). In age-stratified analyses, associations between SES and mortality were much stronger among those aged 70–79 years (*n* = 1086) than among those aged 80 years and older (*n* = 441) (Figure S3).

### Mediating role of health-related behaviours

**Fig. 2:**
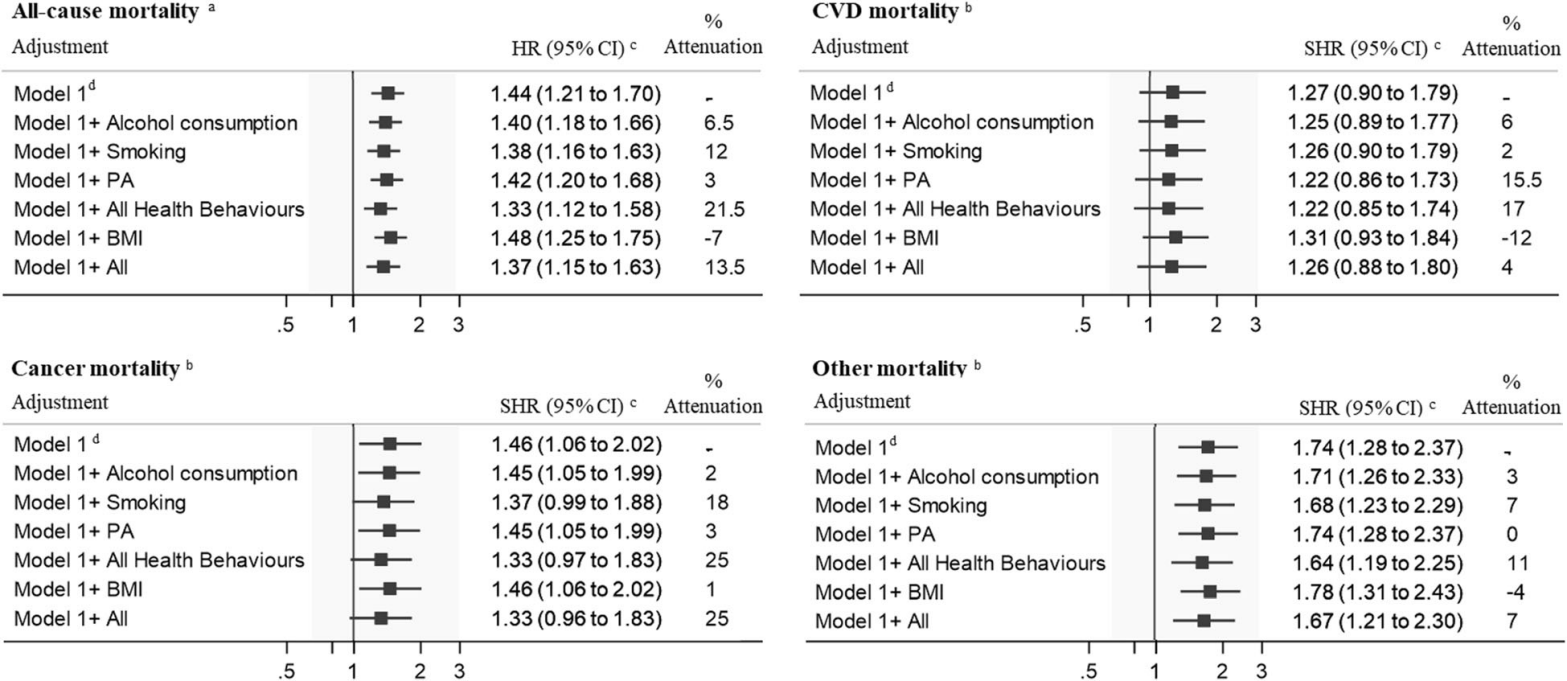
Contribution of health behaviours and body mass index, used as time-dependent covariates, in explaining the association between socioeconomic status and all-cause and cause-specific mortality, the CHAMP study. Abbreviations: HR, hazard ratio; SHR, sub-hazard ratio; PA, physical activity; BMI, body mass index. *N* = 1527. For all-cause mortality, repeated assessment of health-related behaviours, and body mass index at baseline, first, second, and third follow-up were entered into the model as time-varying covariates; for cause-specific mortality repeated assessment at baseline, first, and second follow-up were entered into the model. The study population was divided into three groups by teritles (tertile1: lowest disadvantages-tertile3: highest disadvantages). ^a^ We used calendar year as the time scale, with survivors having a censoring date of 31 December 2017 (person-years follow-up = 13,814). ^b^ We used calendar year as the time scale, with survivors having a censoring date of 31 December 2015 (person-years follow-up = 12,180). ^c^ Hazard ratio and sub-hazard ratio for lowest versus highest tertile (least disadvantages; reference group) of cumulative socioeconomic status. ^d^ Adjusted for age, country of birth, and living arrangement. Percent attenuation =100 × (β_Model1_ − _βModel1 + health behaviour(s)_)/ (β_Model1_), where β = log(Hazard ratio)

No interactions were found between SES indicators and health-related behaviours (all *P*-value> 0.05). Results of analyses of the mediating role of health-related behaviours and BMI in explaining the associations between cumulative SES score and mortality are presented in Fig. 2. For deaths from all causes, the HR for lowest tertile of cumulative SES versus highest tertile (highest socioeconomic status) was 1.44 (95% CI 1.21 to 1.70) in the model adjusted for age, country of birth, and living arrangement. The adjusted SHRs were 1.27 (95% CI 0.90 to 1.79) for CVD mortality, 1.46 (95% CI 1.06 to 2.02) for cancer mortality, and 1.74 (95% CI 1.28 to 2.37) for non-CVD, non-cancer mortality. The associations of cumulative SES with increased all-cause, and non-CVD, non-cancer mortality remained after adjustment for time-varying health behaviours and BMI. Overall, the three health-related behaviours combined explained only 21.5% of the association between cumulative SES score and deaths from all causes; for CVD mortality, the corresponding attenuation was 17%, for cancer mortality 25%, and for non-CVD, non-cancer mortality 11%. As anticipated, smoking contributed the most to the SES inequalities in mortality (ranging between 7 and 18%). The contribution of health-related behaviours to SES-mortality associations decreased after further adjustment for BMI (Fig. 2).

Similar results were apparent regarding the contribution of health-related behaviours to SES inequalities in mortality among participants aged 70–79 years (Table S4). However, among participants aged 80 years and above, health-related behaviours explained 12% of the association between cumulative SES and all-cause mortality compared to 23% in men aged 70–79 years (Table S4).

Results of analyses of the mediating role of health-related behaviours and BMI in explaining associations between each of the four individual SES indicators and mortality are presented in Table S5. The maximum contribution of all health-related behaviours combined was for the association between occupational position and all-cause mortality (27%) and the minimum contribution was for the association between educational attainment and CVD mortality (1%).

In sensitivity analyses testing for reverse-causation, after excluding participants who died during the first two years of follow-up (*n* = 85), the associations between cumulative SES and all-cause and cause-specific mortality remained largely the same as for the whole sample, although the HRs and SHRs were of slightly higher magnitude (Figure S4). The mediating role of health-related behaviours was slightly lower (Figure S4). Overall, all three health-related behaviours combined explained 15% of the SES inequality in deaths from all-causes. In the complete-case analysis, similar effect sizes were evident regarding the association between cumulative SES and mortality from all and specific causes except for cancer mortality, with a slightly weaker association (Figure S5).

## Discussion

In a representative sample of Australian men aged ≥70 years, SES indicators related to early adult life through to older age were predictors of all-cause mortality over 9 years of follow-up. Men who were in low SES groups as assessed by educational attainment, occupational position, and source of income or those who were cumulatively exposed to poor socioeconomic circumstances had higher risk of dying from all causes. We found that associations were stronger among participants aged 70 to 79 years than among those aged ≥80 years. Among the SES indicators used in our analyses, cumulative SES score and source of income were the most consistent predictors of mortality, suggesting that these two are the most relevant SES indicators to mortality at older ages among Australian men. Furthermore, we showed that the role of health-related behaviours (including smoking) in explaining SES gradients in mortality was modest.

Our findings on the educational inequalities in all-cause, and CVD mortality among older men are supported by previous research [4, 5, 7]. Generally, educational attainment is achieved early in adult life, which makes reverse causation implausible—low educational level due to ill health in old age [7]. Higher educational level likely contributes to higher life expectancy through multiple pathways, including healthier behaviours, better adherence to medical treatment, better self-care, and higher occupational positions and incomes [38]. We found no association between educational level and cancer mortality. The nature of the association between educational level and cancer mortality varies by cancer sites [39] but due to small number of deaths from cancer in this study we were unable to analyse SES inequalities for different cancer sites.

Low occupational position was associated with all-cause but not cause-specific mortality. Using occupation as an indicator of SES among older people is debated and previous research among older adults has provided inconsistent evidence concerning occupational position [16, 40–43]. Some argue that occupational position refers to the relatively distant past of older people and does not take into account the specific risks associated with particular occupations [25]. However, in line with our results, several studies have demonstrated persisting occupational inequalities in mortality at older ages [41, 42]. The possible reason for this is that occupational position is an indicator of the position in a social hierarchy, which remain even after leaving paid employment [44].

Source of income was a predictor of all-cause, cancer, and non-CVD, non-cancer mortality. Our result is in line with a study among German men aged ≥65 years that reported significant inequalities in all-cause mortality in related to pension income [42]. Wealth and income inequalities in mortality at older ages have been previously shown in studies mainly from the UK and the US [15, 17, 45]. Our findings of a dose-response association between source of income and deaths from all and specific causes (except for deaths from CVD) shows a higher risk of mortality for Australian men reliant on a government pension solely (a relatively low level of income). This raises the question as to whether governmental policies to increase the level of pension income could lead to reduced mortality rates.

We found no association between housing tenure and all-cause or cause-specific mortality (except for non-CVD, non-cancer mortality), which differs from previous findings in the literature. For instance, one observational study among 31,518 Finnish men aged ≥65 years found significantly higher all-cause mortality among renters compare to owners [46]. In our study, the lack of association between housing tenure and mortality could be due to the fact that most participants were homeowners [47]. This is related to the cultural expectation of homeownership among Australians [25]; in a survey conducted in 2005 and 2006, 75 to 85% of older Australians were homeowners, which is higher than in other affluent countries (https://www.aph.gov.au/Parliamentary_Business/Committees/Senate/Former_Committees/hsaf/report/c02). This result highlights the context dependency of SES [47].

Our finding of the social gradient in all-cause mortality as assessed by a cumulative SES score is consistent with results from the English Longitudinal Study of Aging (ELSA) [18], to our knowledge, the only other study of older people to have used this approach. However, in ELSA, participants with a low relative to high cumulative SES score had a much greater increased risk of CVD mortality (HR of 2.57 (95% CI 1.81–3.65) for both men and women) than we observed in our study (HR of 1.27 (95% CI 0.90–1.79)). Possible explanations for the much stronger association in ELSA include differences in study setting (Australia versus the UK) and sample characteristics, such as mean age of 77.4 years (only men) in our study compared to 65.3 years (both men and women) in ELSA. The methodological differences in cumulative SES score calculation could be another reason; while we used educational attainment, occupational position, source of income, and housing tenure, ELSA used educational attainment, paternal and participant’s own occupation, and wealth. Moreover, the statistical approach to the cumulative SES measure also differed; while we compared lowest (score 5 to 7) versus highest tertile (score 0 to 3) of cumulative SES, ELSA compared individuals who scored 7 or 8 with those scored 0.

Our age-stratified analyses showed that the social gradient in all-cause mortality was stronger among participants aged 70–79 than among those aged ≥80 years. The decline in the strength of the associations between SES inequalities and mortality among participants aged ≥80 years is consistent with previous research that compared SES-mortality associations in middle-aged older adults (< 65 years versus ≥65) [17, 18]. The persisting, albeit weaker, SES gradient in all-cause mortality among the older segment of our sample strengthens the evidence in support of the accumulation hypothesis of disease risk across the life course [28].

Despite the importance of studying health-related behaviours to better understand the socioeconomic inequalities in health and mortality [11, 48], little research has focused on the mediating impact of these factors on SES-mortality associations among older people [15–18]. In this study, the role of health-related behaviours in explaining SES gradients in mortality was modest, which may be due to lack of social patterning of alcohol consumption and physical activity across cumulative SES. For instance, in 2014–2015, only 25% of Australian men aged 65 and over met the guideline of doing 30 min physical activity on 5 or more days and no socioeconomic inequalities were observed for physical activity among older Australian men [49]. Moreover, in Australia, more than 70% of adults aged 70 years and over drink one full serve of alcohol every day, however, they generally drink at levels within the Australian Alcohol Guideline [50]. This result also supports the “age-as-leveler” theory suggesting that the modest contribution of health-related behaviours in SES-mortality inequalities could be due to selective survival of individuals into old age who are relatively resistant to the health consequences of unhealthy behaviours [15]. Previous studies that used individual SES indicators such as wealth, educational level, and occupational position also showed that health-related behaviours failed to completely account for the mortality gradient [15–17]. For instance, in the Health and Retirement Study in the US, health behaviours as assessed at only one point in time accounted for 45% of the wealth-mortality inequalities in men over 65 years and 5% in women [15]. In ELSA, baseline smoking and time-varying physical activity accounted for 31% of the wealth inequalities in mortality at older ages for both men and women [17]. In the only other relevant study that used cumulative SES scores, Stringhini et al., using data from ELSA, reported that smoking, alcohol consumption, physical inactivity, and BMI explained 52% of the association between SES and all-cause mortality [18]. The reason for the differential mediating impact of health-related behaviours in SES inequalities in mortality is probably related to the cultural-dependence of social patterning of these factors [7, 19], as well as demographic characteristic of the participants, between-studies differences in SES measures, and the health-related behaviours examined.

Of the health-related behaviours examined in our study, smoking was the main explanatory factor of the social inequalities in mortality, which agrees with previous literature [3]. The small contribution of alcohol consumption or physical activity to SES inequalities in mortality is because of their weak social patterning [19]. BMI had inverse contribution to SES-mortality gradients, probably partly due to the inverse association between obesity and mortality among older adults [51]. Overall, more than two-thirds of the socioeconomic gradient in mortality remained unexplained after accounting for health-related behaviours and BMI. Other potential mediators of the SES-mortality association are quality of life, material deprivation or financial insecurity, differential access to health care, failure to adhere to medications, psychosocial factors, such as social support and chronic stressors, exposure to environmental risk factors, and exposure to adverse socioeconomic circumstances in utero or during childhood, such as low birth weight and epigenetic modifications [18].

### Strengths and limitations

This study had the benefit of a long follow-up period and high-quality record linkage. A further strength is that the sociodemographic and health-related characteristics of the men in CHAMP are similar to the general population of older men in Australia [52]. The use of repeated measurements of health behaviours and BMI allowed us to consider changes over time.

Limitations of this study merit consideration. The CHAMP study is based on older men and so results may not be applicable to older women or younger adults. Not including individuals living in a residential aged care facility at baseline in CHAMP, who are among the frailer members of society, may potentially underestimate SES-mortality associations. The cultural differences and context-specific characteristics of SES [47] make generalizability of findings more challenging than for biomedical measures. Attrition is a common issue for longitudinal studies of ageing; however, similar results in the imputed and complete-case analyses suggest that this did not influence our results. We did not have data on wealth, one of the important SES measures, particularly at older age [23]. We had data on housing tenure, which does not capture the full accumulated stock of assets. Moreover, we did not have data on working conditions (e.g. job insecurity, long hours, shift work) which might be a better indicator of SES than occupational position among older adults.

Measurement errors are inevitable when subjective measures of health-related behaviours are used which leads to either over or underestimation in the degree of attenuation [53]. Of note, dietary behaviours were only measured at the third follow-up of the CHAMP study; thus, we could not include it as one of the health-related behaviours. In epidemiological studies, there is a debate regarding whether obesity is a health condition or behavioural risk factor [54]. We used BMI as a proxy for nutritional status and diet, as done previously by others [55]. Any unmeasured confounder between health-related behaviours and mortality could have biased the results of the mediation analysis in either direction [56]. Use of the “change in estimate” method is a simple approach regarding mediation analysis which does not consider confounding or interactions between the exposures, mediators, and outcomes [57]. Counterfactual models are an alternative approach [58], but are difficult to apply to survival data with time-dependent mediators [18], and the “change in estimate” method provides a good indication of the existence of indirect effects [59].

Reverse causation between baseline SES and comorbid conditions, is a potential source of bias. However, the results of our sensitivity analyses after excluding those who died the first two years of follow-ups were similar to our main analyses, suggesting that reverse-causation bias is unlikely.

## Conclusion

Our study is one of the first to thoroughly investigate SES inequalities as assessed by four individual determinants of SES as well as cumulative SES score in all-cause and cause-specific mortality among older people. This provided detailed insight into the impact of both individual SES indicators and the cumulative effect of being exposed to adverse SES with mortality. As each indicator of SES influences health through different mechanisms, they are not interchangeable, and our results add to the limited number of studies that evaluated the associations between single indicators of SES and mortality. To our knowledge, this is the first study among older people to examine both individual and cumulative SES inequalities in cancer and non-CVD, non-cancer mortality and the role of repeated measures of health-related behaviours and BMI in explaining SES-mortality association over a long follow-up period. We demonstrated that, in a representative population-based cohort of Australian men aged 70 years and older, low SES, especially source of income and cumulative SES score, was associated with all-cause and cause-specific mortality. Health-related behaviours only partially mediated the SES-mortality associations. These findings support the need for further studies to identify the factors that contribute to SES inequalities in mortality in older people.

## Supplementary information

**Additional file 1 Checklist S1.** STROBE Statement—Checklist of items that should be included in reports of cohort studies. **Table S1.** Missing values of health-related behaviours and body mass index in each follow-up. **Table S2.** Characteristics of the participants included and excluded from the analysis. **Table S3.** Associations between baseline health behaviours and all-cause and cause-specific mortality, the CHAMP study – ANALYSES STRATIFIED BY AGE GROUP. **Table S4.** Role of health-related behaviours, used as time-dependent covariates, in explaining the association between cumulative socioeconomic status score and all-cause and cause-specific mortality, the CHAMP study – ANALYSES STRATIFIED BY AGE GROUP. **Table S5.** Role of health-related behaviours, used as time-dependent covariates, in explaining the association between individual socioeconomic status indicators and all-cause and cause-specific mortality, the CHAMP study. **Figure S1.** Sample restriction flow chart. **Figure S2.** Prevalence of unhealthy behaviours at baseline, first, second, and third follow-up as a function of cumulative SES, the CHAMP study. **Figure S3.** Association of socioeconomic status indicators with all-cause and cause-specific mortality, the CHAMP study - ANALYSES STRATIFIED BY AGE GROUP. **Figure S4.** Contribution of health behaviours and body mass index, used as time-dependent covariates, in explaining the association between socioeconomic status and all-cause and cause-specific mortality, the CHAMP study-AFTER EXCLUDING PARTICIPANTS WHO DIED IN THE FIRST TWO YEARS OF FOLLOEW-UP. **Figure S5.** Contribution of health behaviours and body mass index, used as time-dependent covariates, in explaining the association between socioeconomic status and all-cause and cause-specific mortality, the CHAMP study-complete-case analysis.

## Data Availability

Some access restrictions apply to the data underlying this study’s findings. The original human ethics committee approval for the Concord Health and Ageing in Men Project (CHAMP) in 2004 did not allow for data to be sent outside Australia. Furthermore, the participants in CHAMP have not consented to their data being distributed beyond the CHAMP investigators and their associates. Qualified researchers may submit a request to the CHAMP Management Committee (robert.cumming@sydney.edu.au) and access will require additional ethics approval from the Sydney LHD HREC - CRGH, including considerations of privacy for data sharing.

## Abbreviations

SES: Socioeconomic status
CVD: Cardiovascular disease
PASE: Physical activity scale for the Elderly
BMI: Body mass index
ICD: International Classification of Diseases
