# Supplementary material for: Socioeconomic status, health-related behaviours, and death among older people: the Concord health and aging in men project prospective cohort study
Source: BMC Geriatr. 2020 Jul 29;20:261. doi: 10.1186/s12877-020-01648-y (PMC7391572; doi:10.1186/s12877-020-01648-y)
Supplement: Supplementary file 1 — Additional file 1 Checklist S1. STROBE Statement—Checklist of items that should be included in reports of cohort studies. Table S1. Missing values of health-related behaviours and body mass index in each follow-up. Table S2. Characteristics of the participants included and excluded from the analysis. Table S3. Associations between baseline health behaviours and all-cause and cause-specific mortality, the CHAMP study – ANALYSES STRATIFIED BY AGE GROUP. Table S4. Role of health-related behaviours, used as time-dependent covariates, in explaining the association between cumulative socioeconomic status score and all-cause and cause-specific mortality, the CHAMP study – ANALYSES STRATIFIED BY AGE GROUP. Table S5. Role of health-related behaviours, used as time-dependent covariates, in explaining the association between individual socioeconomic status indicators and all-cause and cause-specific mortality, the CHAMP study. Figure S1. Sample restriction flow chart. Figure S2. Prevalence of unhealthy behaviours at baseline, first, second, and third follow-up as a function of cumulative SES, the CHAMP study. Figure S3. Association of socioeconomic status indicators with all-cause and cause-specific mortality, the CHAMP study - ANALYSES STRATIFIED BY AGE GROUP. Figure S4. Contribution of health behaviours and body mass index, used as time-dependent covariates, in explaining the association between socioeconomic status and all-cause and cause-specific mortality, the CHAMP study-AFTER EXCLUDING PARTICIPANTS WHO DIED IN THE FIRST TWO YEARS OF FOLLOEW-UP. Figure S5. Contribution of health behaviours and body mass index, used as time-dependent covariates, in explaining the association between socioeconomic status and all-cause and cause-specific mortality, the CHAMP study-complete-case analysis. [file 12877_2020_1648_MOESM1_ESM.docx]

**SUPPLEMENTARY INFORMATION**

**Title:** Socioeconomic status, health-related behaviours, and death among older people: The Concord Health and Aging in Men Project prospective cohort study

Saman Khalatbari-Soltani ^1,2^, Fiona M. Blyth ^3^, Vasi Naganathan ^3,4,5^, David J. Handelsman ^6^, David G. Le Couteur ^4,5,6^, Markus J. Seibel ^6^, Louise M. Waite ^3,4,5^, Erin Cvejic^1^, and Robert G. Cumming ^1,2,4^.

^1^The University of Sydney School of Public Health, Faculty of Medicine and Health, New South Wales, Australia; ^2^ARC Centre of Excellence in Population Aging Research (CEPAR), University of Sydney, Sydney, Australia; ^3^Concord Clinical School, Faculty of Medicine and Health, University of Sydney, NSW, Australia; ^4^Centre for Education and Research on Ageing, Faculty of Medicine and Health, University of Sydney, New South Wales, Australia; ^5^Ageing and Alzheimer's Institute, Concord Repatriation and General Hospital, Sydney Local Health District, Concord, New South Wales, Australia; ^6^ANZAC Research Institute, University of Sydney and Concord Hospital, Sydney, Australia.

**Checklist 1.** STROBE Statement—Checklist of items that should be included in reports of *cohort studies*.

|  | Item No | Recommendation |
| --- | --- | --- |
| **Title and abstract** | 1 | (*a*) Indicate the study’s design with a commonly used term in the title or the abstract |
|  |  | (*b*) Provide in the abstract an informative and balanced summary of what was done and what was found |
| Introduction | | |
| Background/rationale | 2 | Explain the scientific background and rationale for the investigation being reported  **Introduction, paragraphs 1 and 2** |
| Objectives | 3 | State specific objectives, including any prespecified hypotheses  **Introduction, paragraph 3** |
| Methods | | |
| Study design | 4 | Present key elements of study design early in the paper  **Methods, paragraph 1** |
| Setting | 5 | Describe the setting, locations, and relevant dates, including periods of recruitment, exposure, follow-up, and data collection  **Methods, paragraph 1** |
| Participants | 6 | (*a*) Give the eligibility criteria, and the sources and methods of selection of participants. Describe methods of follow-up  **Methods, paragraph 1, figure S1, and table S2** |
|  |  | (*b*) For matched studies, give matching criteria and number of exposed and unexposed  **N/A** |
| Variables | 7 | Clearly define all outcomes, exposures, predictors, potential confounders, and effect modifiers. Give diagnostic criteria, if applicable  **Methods (Page 6 to 9)** |
| Data sources/ measurement | 8* | For each variable of interest, give sources of data and details of methods of assessment (measurement). Describe comparability of assessment methods if there is more than one group  **Methods (Page 6 to 9)** |
| Bias | 9 | Describe any efforts to address potential sources of bias  **Methods (Page 9 and 10 statistical analysis)** |
| Study size | 10 | Explain how the study size was arrived at  **Figure S1 & Table S1** |
| Quantitative variables | 11 | Explain how quantitative variables were handled in the analyses. If applicable, describe which groupings were chosen and why  **Methods (Page 6 to 10)** |
| Statistical methods | 12 | (*a*) Describe all statistical methods, including those used to control for confounding  **Methods (Statistical Analysis)** |
|  |  | (*b*) Describe any methods used to examine subgroups and interactions  **Methods (Statistical Analysis-last two paragraph)** |
|  |  | (*c*) Explain how missing data were addressed  **Methods (Statistical analysis, paragraph 1)** |
|  |  | (*d*) If applicable, explain how loss to follow-up was addressed  **Methods (Statistical analysis, paragraph 3)** |
|  |  | (*e*) Describe any sensitivity analyses  **Methods (Statistical analysis, last paragraph)** |
| Results | | |
| Participants | 13* | (a) Report numbers of individuals at each stage of study—eg numbers potentially eligible, examined for eligibility, confirmed eligible, included in the study, completing follow-up, and analysed  **Results, paragraph 1, Figure 1, and table S1** |
|  |  | (b) Give reasons for non-participation at each stage  **Methods, paragraph 1, figure S1, and table S1- we also referred to a previous study that described reasons for non-participation at each follow-up time of the CHAMP study.** |
|  |  | (c) Consider use of a flow diagram  **Figure 1 & table S1** |
| Descriptive data | 14* | (a) Give characteristics of study participants (eg demographic, clinical, social) and information on exposures and potential confounders  **Table 1 and table S2** |
|  |  | (b) Indicate number of participants with missing data for each variable of interest  **Table S1 and figure S1** |
|  |  | (c) Summarise follow-up time (eg, average and total amount)  **Results, paragraph 3 and table 2** |
| Outcome data | 15* | Report numbers of outcome events or summary measures over time  **Results, paragraph 3 and table 2** |
| Main results | 16 | (*a*) Give unadjusted estimates and, if applicable, confounder-adjusted estimates and their precision (eg, 95% confidence interval). Make clear which confounders were adjusted for and why they were included  **Table 2-4, Figure 1, and supplementary Tables and Figures** |
|  |  | (*b*) Report category boundaries when continuous variables were categorized  **Table 2-4, Figure 1, and supplementary Tables and Figures** |
|  |  | (*c*) If relevant, consider translating estimates of relative risk into absolute risk for a meaningful time period  **Table 2 (Age-standardized mortality rate reported)** |
| Other analyses | 17 | Report other analyses done—eg analyses of subgroups and interactions, and sensitivity analyses  **Results and supplementary Tables (S4 & S5) and Figure S4 & S5** |
| Discussion | | |
| Key results | 18 | Summarise key results with reference to study objectives  **Discussion (paragraph 1)** |
| Limitations | 19 | Discuss limitations of the study, taking into account sources of potential bias or imprecision. Discuss both direction and magnitude of any potential bias  **Discussion (pages 18 & 19)** |
| Interpretation | 20 | Give a cautious overall interpretation of results considering objectives, limitations, multiplicity of analyses, results from similar studies, and other relevant evidence  **Discussion** |
| Generalisability | 21 | Discuss the generalisability (external validity) of the study results  **Discussion (Strengths and limitations)** |
| Other information | | |
| Funding | 22 | Give the source of funding and the role of the funders for the present study and, if applicable, for the original study on which the present article is based  **Funding (Page 23)** |

*Give information separately for exposed and unexposed groups.

**Note:** An Explanation and Elaboration article discusses each checklist item and gives methodological background and published examples of transparent reporting. The STROBE checklist is best used in conjunction with this article (freely available on the Web sites of PLoS Medicine at http://www.plosmedicine.org/, Annals of Internal Medicine at http://www.annals.org/, and Epidemiology at http://www.epidem.com/). Information on the STROBE Initiative is available at http://www.strobe-statement.org.

**Table S1.** Missing values of health behaviours and body mass index in each follow-up.

|  | 2-year  Follow-up | | 5-year  Follow-up | 8-year  Follow-up |
| --- | --- | --- | --- | --- |
|  | **Participants** |  |  |  |
|  | n=1248 | | n=884 | n=731 |
| Alcohol consumption | 9 | | 6 | 44 |
| Smoking | 10 | | 2 | 17 |
| Physical activity | 7 | | 4 | 12 |
| Body mass index | 3 | | 22 | 63 |
|  | **Lost to follow-up & alive** | | | |
|  | n=179 | | n=192 | n=90 |

**Table S2.** Characteristics of the participants included and excluded from the analyses.

|  | Included | Excluded | P-value ^a^ |
| --- | --- | --- | --- |
| Characteristic | (n=1527) | (n=178) |  |
| Age, *years* | 77.4±5.5 | 77±5.6 | 0.38 |
| Age categories, % |  |  | 0.98 |
| 70-79 | 71.1 | 70.2 |  |
| 80+ | 28.9 | 28.7 |  |
| Country of birth, % |  |  | 0.002 |
| Australian-born | 51.1 | 38.8 |  |
| Other | 48.9 | 61.2 |  |
| Marital status, % |  |  | 0.86 |
| Single | 5.0 | 5.6 |  |
| Married/Defacto | 76.9 | 75.3 |  |
| Widowed/divorced/separated | 18.1 | 19.1 |  |
| Living alone, % | 18.7 | 18.5 | 0.65 |
| Alcohol consumption, % |  |  | 0.071 |
| Abstainer | 23.1 | 24.7 |  |
| Moderate drinker | 69.0 | 55.1 |  |
| Heavy drinker | 7.9 | 3.4 |  |
| Smoking, % |  |  | 0.41 |
| Non-smoker | 36.8 | 37.6 |  |
| Ex-smoker | 57.2 | 46.6 |  |
| Current smoker | 6.0 | 5.1 |  |
| Physical activity, % |  |  | 0.002 |
| Active | 76.0 | 57.3 |  |
| Inactive | 24.0 | 30.9 |  |
| BMI, kg/m^2^ | 27.8±4 | 28.2±3.9 | 0.17 |
| BMI categories, % |  |  |  |
| Underweight/normal | 24.2 | 19.1 | 0.30 |
| Overweight | 49.0 | 37.6 |  |
| Obese | 26.8 | 27.5 |  |

Data are mean ± SD for continuous variables or percent for categorical variables, unless otherwise stated.

^a^ P-value calculated using Chi-square test for categorical variables and student’s t-test for continuous variables.

Due to some missing data, numbers do not always add to 100%.

**Table S3.** Associations between baseline health-related behaviours and all-cause and cause-specific mortality, the CHAMP study – ANALYSES STRATIFIED BY AGE GROUP.

|  | All-cause mortality | |  | CVD-mortality | |  | Cancer-mortality | |  | Other-mortality  ^a^ | |
| --- | --- | --- | --- | --- | --- | --- | --- | --- | --- | --- | --- |
|  | n | HR ^b^  (95% CI) |  | n | SHR ^b^  (95% CI) |  | n | SHR ^b^  (95% CI) |  | n | SHR ^b^  (95% CI) |
| **70-79 years (n=1086)** |  |  |  |  |  |  |  |  |  |  |  |
| Alcohol consumption |  |  |  |  |  |  |  |  |  |  |  |
| Abstainer (n=226) | 98 | Ref. |  | 13 | Ref. |  | 38 | Ref. |  | 26 | Ref. |
| Moderate drinker (n=765) | 307 | 0.95 (0.75 to 1.19) |  | 52 | 1.22 (0.67 to 2.23) |  | 100 | 0.77 (0.52 to 1.12) |  | 80 | 0.91 (0.58 to 1.41) |
| Heavy drinker (n=95) | 40 | 0.98 (0.67 to 1.43) |  | 11 | 1.97 (0.88 to 4.41) |  | 7 | 0.41 (0.18 to 0.94) |  | 8 | 0.71 (0.32 to 1.58) |
| Smoking |  |  |  |  |  |  |  |  |  |  |  |
| Non-smoker (n=402) | 149 | Ref. |  | 31 | Ref. |  | 38 | Ref. |  | 36 | Ref. |
| Ex-smoker (n=611) | 254 | 1.17 (0.96 to 1.44) |  | 39 | 0.83 (0.52 to 1.32) |  | 92 | 1.63 (1.12 to 2.39) |  | 68 | 1.27 (0.85 to 1.91) |
| Current smoker (n=72) | 42 | 2.21 (1.56 to 3.13) |  | 6 | 1.10 (0.45 to 2.67) |  | 15 | 2.46 (1.33 to 4.53) |  | 10 | 1.63 (0.80 to 3.32) |
| Physical activity |  |  |  |  |  |  |  |  |  |  |  |
| Active (n=873) | 322 | Ref. |  | 53 | Ref. |  | 110 | Ref. |  | 75 | Ref. |
| Inactive (n=212) | 123 | 1.74 (1.41 to 2.14) |  | 23 | 1.74 (1.07 to 2.84) |  | 35 | 1.33 (0.91 to 1.96) |  | 39 | 2.34 (1.58 to 3.46) |
| BMI categories |  |  |  |  |  |  |  |  |  |  |  |
| Underweight/normal (n=227) | 107 | Ref. |  | 21 | Ref. |  | 33 | Ref. |  | 31 | Ref. |
| Overweight (n=535) | 215 | 0.79 (0.63 to 1.00) |  | 36 | 0.74 (0.43 to 1.28) |  | 67 | 0.85 (0.56 to 1.29) |  | 54 | 0.73 (0.46 to 1.13) |
| Obese (n=324) | 123 | 0.74 (0.57 to 0.96) |  | 19 | 0.64 (0.34 to 1.20) |  | 45 | 0.95 (0.60 to 1.49) |  | 29 | 0.64 (0.39 to 1.07) |
|  |  |  |  |  |  |  |  |  |  |  |  |
| **80+ years (n=441)** |  |  |  |  |  |  |  |  |  |  |  |
| Alcohol consumption |  |  |  |  |  |  |  |  |  |  |  |
| Abstainer (n=127) | 104 | Ref. |  | 41 | Ref. |  | 16 | Ref. |  | 38 | Ref. |
| Moderate drinker (n=288) | 213 | 0.79 (0.63 to 1.00) |  | 71 | 0.74 (0.51 to 1.09) |  | 50 | 1.41 (0.80 to 2.48) |  | 58 | 0.63 (0.42 to 0.95) |
| Heavy drinker (n=26) | 21 | 1.01 (0.63 to 1.62) |  | 9 | 1.09 (0.52 to 2.31) |  | 4 | 1.14 (0.39 to 3.34) |  | 8 | 0.94 (0.44 to 2.01) |
| Smoking |  |  |  |  |  |  |  |  |  |  |  |
| Non-smoker (n=160) | 120 | Ref. |  | 51 | Ref. |  | 24 | Ref. |  | 27 | Ref. |
| Ex-smoker (n=261) | 200 | 1.23 (0.98 to 1.55) |  | 64 | 0.77 (0.53 to 1.12) |  | 41 | 1.06 (0.64 to 1.76) |  | 71 | 1.80 (1.15 to 2.82) |
| Current smoker (n=20) | 18 | 2.07 (1.25 to 3.42) |  | 6 | 0.92 (0.39 to 2.14) |  | 5 | 1.82 (0.70 to 4.74) |  | 6 | 2.16 (0.89 to 5.24) |
| Physical activity |  |  |  |  |  |  |  |  |  |  |  |
| Active (n=287) | 208 | Ref. |  | 67 | Ref. |  | 51 | Ref. |  | 57 | Ref. |
| Inactive (n=154) | 130 | 1.52 (1.21 to 1.91) |  | 54 | 1.72 (1.20 to 2.47) |  | 19 | 0.69 (0.41 to 1.17) |  | 47 | 1.76 (1.20 to 2.59) |
| BMI categories |  |  |  |  |  |  |  |  |  |  |  |
| Underweight/normal (n=143) | 117 | Ref. |  | 46 | Ref. |  | 18 | Ref. |  | 41 | Ref. |
| Overweight (n=213) | 159 | 0.82 (0.64 to 1.04) |  | 59 | 0.81 (0.55 to 1.2) |  | 32 | 1.21 (0.67 to 2.18) |  | 45 | 0.72 (0.47 to 1.10) |
| Obese (n=85) | 62 | 0.88 (0.64 to 1.20) |  | 16 | 0.54 (0.30 to 0.95) |  | 20 | 2.03 (1.06 to 3.88) |  | 18 | 0.74 (0.42 to 1.29) |

Abbreviations: CVD, cardiovascular disease; HR, hazard ratio; SHR, sub-hazard ratio.

We used calendar year as the time scale, with survivors having a censoring date of 31 December 2017 (person years follow-up=13814) for all-cause mortality and with survivors having a censoring date of 31 December 2015 (person years follow-up=12180) for cause-specific mortality.

^a^ Indicates non-cancer and non-cardiovascular disease mortality.

^b^ Adjusted for country of birth and living arrangement.

**Table S4.** Role of health-related behaviours, used as time-dependent covariates, in explaining the association between cumulative socioeconomic status score and all-cause and cause-specific mortality, the CHAMP study – ANALYSES STRATIFIED BY AGE GROUP.

|  | 70-79 (n=1086) | |  | 80+ (n=441) | |
| --- | --- | --- | --- | --- | --- |
|  | Hazard ratio ^a^  (95% CI) | %  Attenuation ^b^ |  | Hazard ratio ^a^  (95% CI) | %  Attenuation ^b^ |
| **All-cause mortality** |  |  |  |  |  |
| Model 1 ^c^ | 1.58 (1.27 to 1.97) | - |  | 1.40 (1.07 to 1.85) | - |
| Model 1 + Alcohol | 1.56 (1.25 to 1.94) | 3.5 |  | 1.36 (1.03 to 1.79) | 9.5 |
| Model 1 + Smoking | 1.49 (1.19 to 1.86) | 14 |  | 1.40 (1.06 to 1.85) | 1 |
| Model 1 + Physical activity | 1.52 (1.22 to 1.89) | 9 |  | 1.41 (1.07 to 1.86) | -1.5 |
| Model 1 + All health behaviours | 1.42 (1.14 to 1.78) | 23 |  | 1.35 (1.02 to 1.79) | 12 |
| Model 1 + BMI | 1.61 (1.30 to 2.01) | -4 |  | 1.47 (1.11 to 1.94) | -13 |
| Model 1 + All health behaviours & BMI | 1.44 (1.15 to 1.80) | 20 |  | 1.43 (1.08 to 1.89) | -5 |
|  |  |  |  |  |  |
|  | Sub-Hazard ratio ^a^ (95% CI) | %  Attenuation ^b^ |  | Sub-Hazard ratio ^a^ (95% CI) | %  Attenuation ^b^ |
| **CVD mortality** |  |  |  |  |  |
| Model 1 ^c^ | 1.32 (0.80 to 2.17) | - |  | 1.32 (0.82 to 2.14) | - |
| Model 1 + Alcohol | 1.32 (0.80 to 2.18) | - |  | 1.28 (0.78 to 2.09) | - |
| Model 1 + Smoking | 1.31 (0.79 to 2.18) | - |  | 1.36 (0.84 to 2.21) | - |
| Model 1 + Physical activity | 1.24 (0.75 to 2.07) | - |  | 1.29 (0.80 to 2.09) | - |
| Model 1 + All health behaviours | 1.26 (0.76 to 2.11) | - |  | 1.30 (0.78 to 2.15) | - |
| Model 1 + BMI | 1.32 (0.80 to 2.17) | - |  | 1.42 (0.88 to 2.31) | - |
| Model 1 + All health behaviours & BMI | 1.26 (0.76 to 2.10) | - |  | 1.40 (0.84 to 2.34) | - |
|  |  |  |  |  |  |
| **Cancer mortality** |  |  |  |  |  |
| Model 1 ^c^ | 1.98 (1.34 to 2.93) | - |  | 0.69 (0.36 to 1.31) | - |
| Model 1 + Alcohol | 1.97 (1.34 to 2.90) | 1 |  | 0.68 (0.36 to 1.29) | - |
| Model 1 + Smoking | 1.80 (1.21 to 2.65) | 14.5 |  | 0.68 (0.36 to 1.29) | - |
| Model 1 + Physical activity | 1.94 (1.31 to 2.87) | 3 |  | 0.69 (0.36 to 1.30) | - |
| Model 1 + All health behaviours | 1.73 (1.17 to 2.56) | 20 |  | 0.67 (0.35 to 1.26) | - |
| Model 1 + BMI | 2.00 (1.35 to 2.96) | -1 |  | 0.67 (0.36 to 1.27) | - |
| Model 1 + All health behaviours & BMI | 1.75 (1.18 to 2.60) | 18 |  | 0.66 (0.35 to 1.23) | - |
|  |  |  |  |  |  |
| **Other mortality** ^d^ |  |  |  |  |  |
| Model 1 ^c^ | 2.10 (1.38 to 3.20) | - |  | 1.50 (0.94 to 2.40) | - |
| Model 1 + Alcohol | 2.07 (1.36 to 3.16) | 2 |  | 1.45 (0.90 to 2.32) | - |
| Model 1 + Smoking | 2.01 (1.29 to 3.11) | 6 |  | 1.49 (0.92 to 2.39) | - |
| Model 1 + Physical activity | 2.06 (1.35 to 3.12) | 3 |  | 1.52 (0.96 to 2.43) | - |
| Model 1 + All health behaviours | 2.00 (1.29 to 3.10) | 7 |  | 1.43 (0.89 to 2.30) | - |
| Model 1 + BMI | 2.14 (1.40 to 3.27) | -3 |  | 1.54 (0.96 to 2.48) | - |
| Model 1 + All health behaviours & BMI | 2.05 (1.32 to 3.19) | 3 |  | 1.45 (0.90 to 2.33) | - |

Abbreviation: BMI, body mass index.

N=1527. Of 1086 participants aged 70 to 79 years, there were 445, 76, 145, and 114 deaths attributable to all-cause, CVD, cancer, and non-cancer, non-CVD mortality; and of the 441 participants aged 80 years and above, there were 338, 121, 70, and 104 deaths attributable to all-cause, CVD, cancer, and non-cancer, non-CVD mortality.

For all-cause mortality, repeated assessment of health-related behaviours, and body mass index at baseline, first, second, and third follow-up were entered into the model as time-varying covariates; for cause-specific mortality repeated assessment of baseline, first, and second follow-up were entered into the model.

We used calendar year as the time scale, with survivors having a censoring date of 31 December 2017 (person years follow-up=13814) for all-cause mortality and with survivors having a censoring date of 31 December 2015 (person years follow-up=12180) for cause-specific mortality.

The study population was divided into three groups by teritles (tertile1: lowest disadvantages-tertile3: highest disadvantages).

^a^ Hazard ratio for lowest versus highest tertile (least disadvantages; reference model) of cumulative socioeconomic status.

^b^ Percent attenuation =100×(β_Model1_−β_Model1+health behaviour(s)_)/ (β_Model1_), where β =log(Hazard ratio). Percent attenuation is calculated only for statistically significant associations.

^c^ Adjusted for country of birth and living arrangement.

^d^ Indicates non-cardiovascular disease and non-cancer mortality.

**Table S5.** Role of health-related behaviours, used as time-dependent covariates, in explaining the association between individual socioeconomic status indicators and all-cause and cause-specific mortality, the CHAMP study.

|  | Educational attainment | | Occupational position | | Sources of income | | Housing tenure | |
| --- | --- | --- | --- | --- | --- | --- | --- | --- |
|  | Hazard ratio ^a^  (95% CI) | %Attenuation ^b^ | Hazard ratio^a^  (95% CI) | %Attenuation ^b^ | Hazard ratio^a^  (95% CI) | %Attenuation ^b^ | Hazard ratio^a^  (95% CI) | %Attenuation ^b^ |
| **All-cause mortality** |  |  |  |  |  |  |  |  |
| Model 1 ^c^ | 1.41 (1.10, 1.80) |  | 1.20 (1.00 to 1.45) | - | 1.44 (1.23 to 1.70) | - | 1.18 (0.94 to 1.47) | - |
| Model 1 + Alcohol | 1.41 (1.11 to 1.80) | -1 | 1.18 (0.99 to 1.42) | 9 | 1.42 (1.21 to 1.68) | 4 | 1.15 (0.92 to 1.44) | - |
| Model 1 + Smoking | 1.35 (1.06 to 1.73) | 11 | 1.19 (0.99 to 1.43) | 7 | 1.40 (1.19 to 1.65) | 8 | 1.17 (0.94 to 1.45) | - |
| Model 1 + Physical activity | 1.38 (1.08 to 1.76) | 6.5 | 1.18 (0.98 to 1.41) | 12 | 1.47 (1.24 to 1.73) | -4 | 1.09 (0.87 to 1.36) | - |
| Model 1 + All health behaviours | 1.33 (1.04 to 1.70) | 17 | 1.14 (0.95 to 1.38) | 27 | 1.41 (1.19 to 1.66) | 7 | 1.06 (0.85 to 1.32) | - |
| Model 1 + BMI | 1.50 (1.17 to 1.92) | -18 | 1.23 (1.02 to 1.48) | -12 | 1.47 (1.25 to 1.73) | -4 | 1.14 (0.91 to 1.42) | - |
| Model 1 + All health behaviours & BMI | 1.41 (1.11 to 1.81) | -1 | 1.17 (0.97 to 1.41) | 14 | 1.43 (1.21 to 1.69) | 3 | 1.03 (0.82 to 1.28) | - |
|  |  |  |  |  |  |  |  |  |
|  | Sub-Hazard ratio^a^ (95% CI) | %Attenuation ^b^ | Sub-Hazard ratio^a^ (95% CI) | %Attenuation ^b^ | Sub-Hazard ratio^a^ (95% CI) | %Attenuation ^b^ | Sub-Hazard ratio^a^ (95% CI) | %Attenuation ^b^ |
| **CVD mortality** |  |  |  |  |  |  |  |  |
| Model 1 ^c^ | 1.75 (1.04 to 2.93) | - | 1.28 (0.88 to 1.87) | - | 1.11 (0.81 to 1.54) | - | 0.86 (0.53 to 1.39) | - |
| Model 1 + Alcohol | 1.75 (1.04 to 2.93) | 0 | 1.27 (0.87 to 1.86) | - | 1.11 (0.80 to 1.53) | - | 0.85 (0.52 to 1.38) | - |
| Model 1 + Smoking | 1.78 (1.07 to 2.98) | -3.5 | 1.28 (0.87 to 1.87) | - | 1.10 (0.79 to 1.51) | - | 0.86 (0.53 to 1.38) | - |
| Model 1 + Physical activity | 1.70 (1.01 to 2.87) | 5 | 1.24 (0.84 to 1.82) | - | 1.12 (0.81 to 1.55) | - | 0.76 (0.46 to 1.24) | - |
| Model 1 + All health behaviours | 1.74 (1.03 to 2.93) | 1 | 1.23 (0.84 to 1.81) | - | 1.11 (0.80 to 1.53) | - | 0.75 (0.46 to 1.24) | - |
| Model 1 + BMI | 1.88 (1.12 to 3.14) | -13 | 1.28 (0.88 to 1.87) | - | 1.16 (0.83 to 1.60) | - | 0.84 (0.52 to 1.35) | - |
| Model 1 + All health behaviours & BMI | 1.87 (1.11 to 3.15) | -12 | 1.24 (0.84 to 1.83) | - | 1.15 (0.83 to 1.59) | - | 0.73 (0.45 to 1.20) | - |
|  |  |  |  |  |  |  |  |  |
| **Cancer mortality** |  |  |  |  |  |  |  |  |
| Model 1 ^c^ | 1.21 (0.76 to 1.92) | - | 1.30 (0.92 to 1.85) | - | 1.58 (1.16 to 2.15) | - | 1.02 (0.67 to 1.56) | - |
| Model 1 + Alcohol | 1.22 (0.76 to 1.94) | - | 1.29 (0.91 to 1.83) | - | 1.57 (1.15 to 2.13) | 2 | 1.00 (0.65 to 1.53) | - |
| Model 1 + Smoking | 1.13 (0.71 to 1.79) | - | 1.28 (0.90 to 1.82) | - | 1.50 (1.10 to 2.05) | 11 | 1.00 (0.66 to 1.53) | - |
| Model 1 + Physical activity | 1.20 (0.75 to 1.91) | - | 1.30 (0.91 to 1.85) | - | 1.58 (1.16 to 2.15) | 0.5 | 0.97 (0.64 to 1.48) | - |
| Model 1 + All health behaviours | 1.12 (0.71 to 1.78) | - | 1.26 (0.89 to 1.78) | - | 1.48 (1.09 to 2.02) | 14 | 0.94 (0.61 to 1.43) | - |
| Model 1 + BMI | 1.21 (0.76 to 1.93) | - | 1.30 (0.91 to 1.85) | - | 1.58 (1.16 to 2.15) | 0 | 1.01 (0.66 to 1.55) | - |
| Model 1 + All health behaviours & BMI | 1.12 (0.71 to 1.79) | - | 1.25 (0.88 to 1.78) | - | 1.48 (1.09 to 2.02) | 14 | 0.93 (0.61 to 1.43) | - |
|  |  |  |  |  |  |  |  |  |
| **Other mortality**  ^d^ |  |  |  |  |  |  |  |  |
| Model 1 ^c^ | 1.31 (0.81 to 2.10) | - | 1.11 (0.79 to 1.57) | - | 1.70 (1.25 to 2.30) | - | 1.60 (1.09 to 2.35) | - |
| Model 1 + Alcohol | 1.30 (0.81 to 2.09) | - | 1.09 (0.78 to 1.55) | - | 1.68 (1.24 to 2.28) | 3 | 1.58 (1.08 to 2.32) | 3 |
| Model 1 + Smoking | 1.21 (0.75 to 1.96) | - | 1.09 (0.77 to 1.54) | - | 1.66 (1.22 to 2.26) | 4 | 1.58 (1.08 to 2.32) | 2 |
| Model 1 + Physical activity | 1.27 (0.79 to 2.05) | - | 1.11 (0.78 to 1.57) | - | 1.73 (1.27 to 2.35) | -3 | 1.49 (1.02 to 2.17) | 15 |
| Model 1 + All health behaviours | 1.17 (0.73 to 1.90) | - | 1.07 (0.76 to 1.52) | - | 1.66 (1.21 to 2.27) | 4.5 | 1.46 (1.00 to 2.13) | 20 |
| Model 1 + BMI | 1.40 (0.86 to 2.27) | - | 1.14 (0.81 to 1.62) | - | 1.71 (1.26 to 2.32) | -1 | 1.58 (1.08 to 2.32) | 3 |
| Model 1 + All health behaviours & BMI | 1.25 (0.77 to 2.03) | - | 1.11 (0.78 to 1.58) | - | 1.67 (1.22 to 2.29) | 3 | 1.43 (0.98 to 2.09) | 24 |

Abbreviation: BMI, body mass index.

N=1527.

Educational attainment was categorized as ‘high’ (university degree), ‘intermediate’ (trade, apprenticeship, certificate, or diploma), and ‘low’ (no post-school qualification); Occupational level was categorized as ‘high’ (higher professional and managers, lower professionals and managers, higher clerical services and sales workers), ‘intermediate’ (small employers and self-employed, farmers, lower supervisors and technicians), and ‘low’ (lower clerical, services, sales workers, skilled and unskilled workers); and income was categorized as ‘high’ (other sources of income only), ‘intermediate’ (reliant on a government pension plus other source of income), and ‘low’ (reliant on a government pension only).

For all-cause mortality, repeated assessment of health-related behaviours, and body mass index at baseline, first, second, and third follow-up were entered into the model as time-varying covariates; for cause-specific mortality repeated assessment of baseline, first, and second follow-up were entered into the model.

We used calendar year as the time scale, with survivors having a censoring date of 31 December 2017 (person years follow-up=13814) for all-cause mortality and with survivors having a censoring date of 31 December 2015 (person years follow-up=12180) for cause-specific mortality.

^a^ Hazard ratio for lowest versus highest tertile (least disadvantages; reference model) of cumulative socioeconomic status.

^b^ Percent attenuation =100×(β_Model1_−β_Model1+health behaviour(s)_)/ (β_Model1_), where β =log(Hazard ratio). Percent attenuation is calculated only for statistically significant associations.

^C^ Adjusted for age, country of birth, and living arrangement.

^d^ Indicates non-cardiovascular disease and non-cancer mortality.

# **Figure S1.** Sample selection flow chart.

Disagreement with mortality data linkage

(n=66, 3.9%)

Total original sample (n=1705)

Missing socioeconomic data (n=55, 3.2%)

Total analytic sample (n=1527, 89.6%)

Missing modifiable risk factors (n=55, 2.2%) or covariates (n=2, 0.1%)

**Figure S2.** Prevalence of unhealthy behaviours at baseline, first, second, and third follow-up as a function of cumulative SES, the CHAMP study.


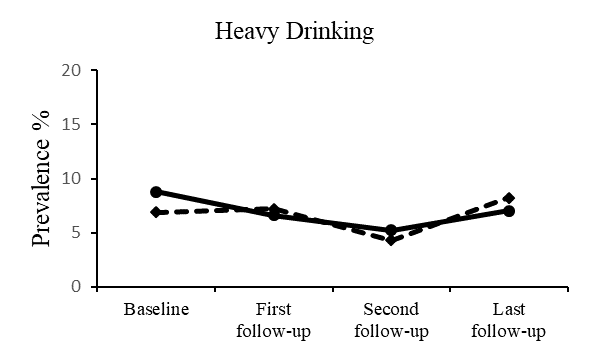

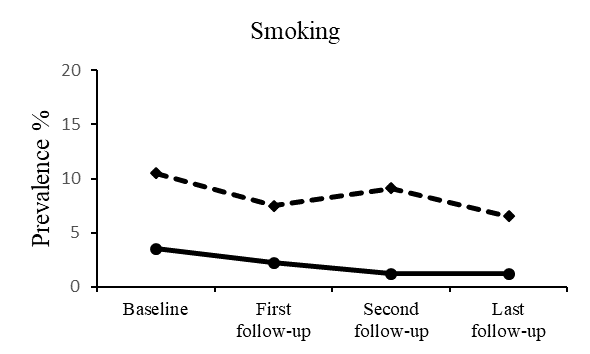

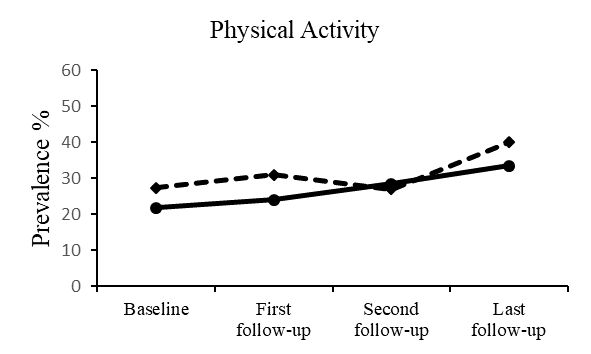

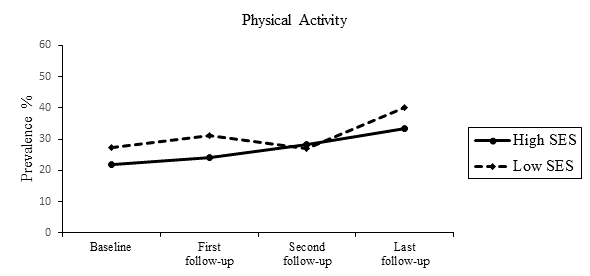


**70-79+** (n=1086) **+**

**All-cause mortality**

**CVD mortality**

**Cancer mortality**

**Other mortality** ^a^

**80+** (n=441)

P_trend_ <0.001

P_trend_ =0.004

P_trend_ =0.019

P_trend_ <0.001

P_trend_ =0.28

P_trend_ =0.11

P_trend_ =0.49

P_trend_ =0.28

P_trend_ <0.001

P_trend_ =0.14

P_trend_ =0.10

P_trend_ <0.001

P_trend_ <0.001

P_trend_ =0.18

P_trend_ =0.41

P_trend_ <0.001

P_trend_ =0.013

P_trend_ =0.21

P_trend_ =0.046

P_trend_ =0.29

P_trend_ =0.66

P_trend_ =0.42

P_trend_ =0.85

P_trend_ =0.72

P_trend_ =0.50

P_trend_ =0.88

P_trend_ =0.16

P_trend_ =0.04

P_trend_ =0.25

P_trend_ =0.25

P_trend_ =0.091

P_trend_ =0.10

SHR (95% CI)

SES indicator

Deaths

Deaths

Deaths

Deaths

HR (95% CI)

SHR (95% CI)

SHR (95% CI)

**Figure S3.** Association of socioeconomic status indicators with all-cause and cause-specific mortality, the CHAMP study - ANALYSES STRATIFIED BY AGE GROUP.

Abbreviations: CVD, cardiovascular disease; SES, socioeconomic status; HR, hazard ratio; SHR, sub-hazard ratio.

We used calendar year as the time scale, with survivors having a censoring date of 31 December 2017 (person-years follow-up=13814) for all-cause mortality and with survivors having a censoring date of 31 December 2015 (person-years follow-up=12180) for cause-specific mortality.

All estimates were adjusted for country of birth and living arrangement.

^a^ Indicates non-cardiovascular disease and non-cancer mortality.

Adjustment

% Attenuation

HR

(95% CI) ^c^

**All-cause mortality** ^a^

**CVD mortality** ^b^

**Cancer mortality** ^b^

**Other mortality** ^b^

Adjustment

% Attenuation

SHR

(95% CI) ^c^

Adjustment

% Attenuation

SHR

(95% CI) ^c^

Adjustment

% Attenuation

SHR

(95% CI) ^c^

-

-

-

-

^d^

^d^

^d^

^d^

**Figure S4.** Contribution of health behaviours and body mass index, used as time-dependent covariates, in explaining the association between socioeconomic status and all-cause and cause-specific mortality, the CHAMP study-AFTER EXCLUDING PARTICIPANTS WHO DIED IN THE FIRST TWO YEARS OF FOLLOEW-UP.

Abbreviations: HR, hazard ratio; SHR, sub-hazard ratio; PA, physical activity; BMI, body mass index.

N=1442 after excluding 85 participants who died the first two years of follow-up.

For all-cause mortality, repeated assessment of health-related behaviours, and body mass index at baseline, first, second, and third follow-up were entered into the model as time-varying covariates; for cause-specific mortality repeated assessment at baseline, first, and second follow-up were entered into the model.

The study population was divided into three groups by teritles (tertile1: lowest disadvantages-tertile3: highest disadvantages).

^a^ We used calendar year as the time scale, with survivors having a censoring date of 31 December 2017 (person-years follow-up=13814).

^b^ We used calendar year as the time scale, with survivors having a censoring date of 31 December 2015 (person-years follow-up=12180).

^c^ Hazard ratio and sub-hazard ratio for lowest versus highest tertile (least disadvantages; reference group) of cumulative socioeconomic status.

^d^ Adjusted for age, country of birth, and living arrangement.

Percent attenuation =100×(β_Model1_−β_Model1+health behaviour(s)_)/ (β_Model1_), where β =log(Hazard ratio).

Adjustment

% Attenuation

HR

(95% CI) ^c^

**All-cause mortality** ^a^

**CVD mortality**^b^

**Other mortality**^b^

Adjustment

% Attenuation

SHR

(95% CI) ^c^

Adjustment

% Attenuation

SHR

(95% CI) ^c^

Adjustment

% Attenuation

SHR

(95% CI) ^c^

-

**Cancer mortality**^b^

-

-

-

-

-

-

-

-

-

-

-

-

-

-

-

^d^

^d^

^d^

^d^

**Figure S5.** Contribution of health behaviours and body mass index, used as time-dependent covariates, in explaining the association between socioeconomic status and all-cause and cause-specific mortality, the CHAMP study-complete-case analysis.

Abbreviations: HR, hazard ratio; SHR, sub-hazard ratio; PA, physical activity; BMI, body mass index.

N=939.

For all-cause mortality, repeated assessment of health-related behaviours, and body mass index at baseline, first, second, and third follow-up were entered into the model as time-varying covariates; for cause-specific mortality repeated assessment at baseline, first, and second follow-up were entered into the model.

The study population was divided into three groups by teritles (tertile1: lowest disadvantages-tertile3: highest disadvantages).

^a^ We used calendar year as the time scale, with survivors having a censoring date of 31 December 2017 (person-years follow-up=13814).

^b^ We used calendar year as the time scale, with survivors having a censoring date of 31 December 2015 (person-years follow-up=12180).

^c^ Hazard ratio and sub-hazard ratio for lowest versus highest tertile (least disadvantages; reference group) of cumulative socioeconomic status.

^d^ Adjusted for age, country of birth, and living arrangement.

Percent attenuation =100×(β_Model1_−β_Model1+health behaviour(s)_)/ (β_Model1_), where β =log(Hazard ratio).
